# Strain Selection for Generation of O-Antigen-Based Glycoconjugate Vaccines against Invasive Nontyphoidal *Salmonella* Disease

**DOI:** 10.1371/journal.pone.0139847

**Published:** 2015-10-07

**Authors:** Luisa Lanzilao, Giuseppe Stefanetti, Allan Saul, Calman A. MacLennan, Francesca Micoli, Simona Rondini

**Affiliations:** 1 Sclavo Behring Vaccines Institute for Global Health S.r.l., a GSK company (formerly Novartis Vaccines Institute for Global Health S.r.l), Via Fiorentina 1, 53100, Siena, Italy; 2 Jenner Institute, Nuffield Department of Medicine, University of Oxford, Old Road Campus Research Building, Roosevelt Drive, Oxford, OX3 7DQ, United Kingdom; 3 Wellcome Trust Sanger Institute, Wellcome Trust Genome Campus, Hinxton, Cambridge, CB10 1SA, United Kingdom; New York State Dept. Health, UNITED STATES

## Abstract

Nontyphoidal *Salmonellae*, principally *S*. Typhimurium and *S*. Enteritidis, are a major cause of invasive bloodstream infections in sub-Saharan Africa with no vaccine currently available. Conjugation of lipopolysaccharide O-antigen to a carrier protein constitutes a promising vaccination strategy. Here we describe a rational process to select the most appropriate isolates of *Salmonella* as source of O-antigen for developing a bivalent glycoconjugate vaccine. We screened a library of 30 *S*. Typhimurium and 21 *S*. Enteritidis in order to identify the most suitable strains for large scale O-antigen production and generation of conjugate vaccines. Initial screening was based on growth characteristics, safety profile of the isolates, O-antigen production, and O-antigen characteristics in terms of molecular size, O-acetylation and glucosylation level and position, as determined by phenol sulfuric assay, NMR, HPLC-SEC and HPAEC-PAD. Three animal isolates for each serovar were identified and used to synthesize candidate glycoconjugate vaccines, using CRM_197_ as carrier protein. The immunogenicity of these conjugates and the functional activity of the induced antibodies was investigated by ELISA, serum bactericidal assay and flow cytometry. *S*. Typhimurium O-antigen showed high structural diversity, including O-acetylation of rhamnose in a Malawian invasive strain generating a specific immunodominant epitope. *S*. Typhimurium conjugates provoked an anti-O-antigen response primarily against the O:5 determinant. O-antigen from *S*. Enteritidis was structurally more homogeneous than from *S*. Typhimurium, and no idiosyncratic antibody responses were detected for the *S*. Enteritidis conjugates. Of the three initially selected isolates, two *S*. Typhimurium (1418 and 2189) and two *S*. Enteritidis (502 and 618) strains generated glycoconjugates able to induce high specific antibody levels with high breadth of serovar-specific strain coverage, and were selected for use in vaccine production. The strain selection approach described is potentially applicable to the development of glycoconjugate vaccines against other bacterial pathogens.

## Introduction

Bacteria of the species *Salmonella enterica* causing human disease are divided into human-restricted typhoidal serovars (Typhi and Paratyphi) causing enteric fever, and nontyphoidal *Salmonella* (NTS) serovars, which have a broader host-range and are frequently zoonotic. Among the NTS serovars isolated worldwide from humans, *Salmonella* Enteritidis and Typhimurium rank as the most common (43.5%) and second most common (17.1%), respectively [1]. In developed countries NTS cause a mild self-limiting gastroenteritis with less than 2% mortality. In those countries, invasive nontyphoidal *Salmonella* (iNTS) disease is uncommon [2], with an estimated overall crude annual incidence of 49/100,000 population [3]. In contrast, in sub-Saharan Africa, *S*. Typhimurium and Enteritidis are the predominant cause of invasive bloodstream infections, especially amongst young children and HIV-infected individuals [4, 5]. The case-fatality rate of iNTS disease is up to 25% and the effectiveness of antibiotic treatment has been hampered by increasing multidrug resistance [4, 5].

Currently, there are no licensed vaccines against iNTS disease and efforts are ongoing to identify protective antigens and best strategies for vaccine development [4, 6, 7]. Lipopolysaccharide (LPS) has been implicated as a target of the protective immune response [8–11] and the serovar-specific O-antigen (OAg) of LPS have been employed in subunit vaccines, particularly glycoconjugate vaccines. OAg have been shown to elicit protective immunity against lethal challenge, with anti-OAg antibodies protective in adoptive transfer experiments [12–16]. It is known that OAg expression is regulated by several factors including serum exposure [17], growth phase [18], presence of micronutrients [17, 19, 20]. OAg length and density can determine bacterial pathogenicity [18, 21–23]. Bacterial LPS can demonstrate high levels of heterogeneity: OAg can vary in chain length and can have other modifications, such as glucosylation and O-acetylation of sugar residues in the repeating units [24–27]. Little is known about the influence of these OAg modifications on pathogenicity [27], and how OAg can vary among strains belonging to the same *S*. Typhimurium and Enteritidis serovar. As the variety of LPS modifications occurring in nature is not known, it is unclear whether these differences are critical for the development of an OAg-based vaccine, and what importance should be attached to selecting the OAg source.

Previous work [11] has shown that the highest anti-OAg antibody responses elicited by *S*. Typhimurium OAg-CRM_197_ conjugates are obtained from OAg with the highest glucosylation levels, OAg populations of medium (approximately 25 repeating OAg units) or mixed molecular weights (composed of both medium and high molecular weights: around 70 OAg repeating units) with an optimal OAg/CRM_197_ ratio of 1.5. Additionally, the interplay between glucosylation and O-acetylation appears to be important in eliciting bactericidal anti-OAg antibodies. Based on these findings, we screened a library of 30 *S*. Typhimurium and 21 Enteritidis, with the specific aim of identifying those most suitable for large scale OAg production and generation of OAg-conjugate vaccines cross-protective against endemic invasive African strains. The criteria used to down-select these strains were based on OAg production levels, safety (antibiotic susceptibility and known invasiveness) and recognition of antibodies generated against an endemic invasive African isolate. At the conclusion of this selection process, we were able to identify three strains for each serovar, all amenable to large-scale manufacture, and, in the case of *S*. Typhimurium, having different OAg features in terms of molecular size, O-acetylation and glucosylation level and position. We used the OAg from these strains to generate conjugate vaccines and evaluated the impact of OAg structure on immunogenicity. Our findings can help guide the selection of the most suitable OAg-producing strains for effective vaccines against iNTS disease.

## Results

### Strains Screening and Selection

The first criterion for NTS strain selection was based on the evaluation of their growth characteristics. All 51 NTS isolates (30 *S*. Typhimurium and 21 *S*. Enteritidis), were inoculated in Chemically Defined (CD) medium and grown overnight. We chose CD medium) as a suitable growth medium for large-scale production [28]. Twenty-seven *S*. Typhimurium and 19 *S*. Enteritidis grew well reaching Optical Densities (ODs) of 3–6; 3 *S*. Typhimurium did not grow unless the medium was supplemented with casamino acids, while 2 *S*. Enteritidis grew poorly in CD medium reaching ODs of 1–1.5 (Tables 1 and 2). From this initial screening, only the isolates that grew to OD>3 in CD medium (27 *S*. Typhimurium and 19 *S*. Enteritidis) were selected for further analysis.

**Table 1:** *S*. Typhimurium isolates.

Isolate N.	Original collection/ identification	Source	Country	Growth	OAg production (μg/mL)	OAg-producer	Antibiotic resistance	avMW OAg (KDa)*	O-acetylation	Fluorescent signal**
1415	SGSC, LT5	Calf	Zurich, Switzerland	CD medium	436	Medium	S	-	-	-
1416	SGSC, LT6	Pigeon	Visby, Sweden	CD medium	735	Medium	S	-	-	-
1417	SGSC, LT7	Lamb	Fort Collins, USA	CD medium	1300	High	S	68.7 and 28,3	Abequose	2322
1418	SGSC, LT8	Mouse	Denmark	CD medium	1560	High	S	87 and 30.4	Abequose	2202
1419	SGSC, LT9	Turkey	Minnesota, USA	CD medium	1189	High	Aztreonam	-	-	-
1421	SGSC, LT11	Rat	Sweden	CD medium	915	High	S	63.8 and 28.3	Abequose, Rhamnose	2442
1429	SGSC, LT19	Human	Surahammar, Sweden	CD medium	696	Medium	S	-	-	-
1430	SGSC, LT20	Hen	Chile	CD medium	800	-	S	-	-	-
2181	SGSC, SARA 1	Human	Mexico	CD medium	461	Medium	S	-	-	-
2183	SGSC, SARA 3	Horse	Rhode Island	CD medium	22	Low	Aztreonam, chloramphenicol	-	-	-
2184	SGSC, SARA 4	Rabbit	Indiana	CD medium	106	Low	Ampicillin	-	-	-
2185	SGSC, SARA 5	Rabbit	Mongolia	CD medium	684	Medium	S	-	-	-
2186	SGSC, SARA 6	Human	Ohio	CD medium	133	Low	S	-	-	-
2187	SGSC, SARA 7	Human	Norway	Supplements	1098	-	S	-	-	-
2188	SGSC, SARA 8	Human	Finland	CD medium	561	Medium	S	-	-	-
2189	SGSC, SARA 9	Parrot	California	CD medium	990	High	S	80.8 and 31.6	Abequose	268
2190	SGSC, SARA 10	Opossum	California	CD medium	47	Low	S	-	-	-
2191	SGSC, SARA 11	Opossum	Thailand	CD medium	790	Medium	S	-	-	-
2192	SGSC, SARA 12	Horse	Louisiana	CD medium	1172	High	S	34.5	Abequose	959
2193	SGSC, SARA 13	Horse	France	CD medium	864	High	S	< 5.5	Abequose	807
2194	SGSC, SARA 14	Horse	Panama	Supplements	784	-	S	-	-	-
2195	SGSC, SARA 15	Dog	Texas	CD medium	567	Medium	S	-	-	-
2196	SGSC, SARA 16	Human	North Carolina	CD medium	1400	High	S	-	-	-
2197	SGSC, SARA 17	Human	Yugoslavia	CD medium	508	Medium	S	-	-	-
2198	SGSC, SARA 18	Horse	Iowa	CD medium	937	High	S	85.7 and 30.4	Abequose	2927
2199	SGSC, SARA 19	Human	Mexico	Supplements	924	-	S	-	-	-
2200	SGSC, SARA 20	Human	France	CD medium	370	Low	S	-	-	-
2201	SGSC, SARA 21	Heron	Oregon	CD medium	312	Low	S	-	-	-
SA5983	Lab of Foodborne Zoonoses Health	Chicken	Canada	CD medium	556	Medium	S	-	-	-
SA5984	Lab of Foodborne Zoonoses Health	Pig	Canada	CD medium	476	Medium	S	-	-	-
D23580	Malawi-Liverpool-Wellcome Trust Clinical Research Programme	Human, invasive isolate	Malawi	CD medium	699	Medium	Chloramphenicol, ampicillin, kanamycin, streptomycin, sulphonamid, trimethoprim	82.6 and 28.3	Abequose, Rhamnose	-
LT2	Novartis Master Culture Collection	Human, lab strain	UK	CD medium	1021	High	S	82.6 and 28.3	Abequose	-
SL1344	University of Birmingham	Calf, laboratory strain	UK	CD medium	702	High	S	82.6 and 28.3	Abequose	-

SGSC: *Salmonella* Genetic Stock CentreSARA: *Salmonella* reference collection A. CD: Chemically DefinedS: sensitive to ampicillin, amoxicillin, aztreonam, cefotaxime, ceftazidime, ceftriaxone, ciprofloxacin, chloramphenicol, cotrimoxazole, meropenem

*avMW: average molecular weight, as determined by HLP-SEC

** values represent the geometric mean of the fluorescent signal as determined by FACS

**Table 2:** *S*. Enteritidis isolates.

Isolate N.	Original collection/identification	Source	Country	Growth	OAg production (μg/mL)	OAg-producer	Antibiotic resistance	avMW OAg (KDa)*	O-acetylation	Fluorescent signal**
IV3452001	EASSA 3;M2008-10019928	Chicken	Hungary	CD medium	873	High	S	32.4	-	-
IV3452067	EASSA 3;M2008-10022324	Chicken	Hungary	CD medium	1005	High	S	<5.5	-	-
IV3453219	EASSA 3;PL005174953749	Cattle	Poland	CD medium	845	Medium	S	82.6 and 30.5	Present	1169
IV3456065	EASSA 3;09CEB11144SAL	Pig	France	CD medium	443	Medium	S	80.0 (shoulder) and 34.4	-	-
IV3456074	EASSA 3;09CEB495SAL	Chicken	France	CD medium	685	Medium	S	34.4	Present	1958
384	SA, EASSA 2;E187	Chicken	France	CD medium	585	Medium	-	32.6	-	-
386	SA, EASSA 2;I51	Chicken	France	CD medium	862	High	-	32.6	-	-
502	SA, EASSA 2;salm46962	Chicken	Germany	CD medium	740	Medium	S	77 and 30.5	Present	1373
505	SA, EASSA 2;salm50830	Chicken	Germany	CD medium	562	Medium	-	32.6	-	-
506	SA, EASSA 2;salm50157	Chicken	Germany	CD medium	841	Medium	-	32.6	Present	1118
507	SA, EASSA 2;salm50337	Chicken	Germany	CD medium	722	Medium	-	32.6	-	-
596	SA, EASSA 2;P3S245-247	Chicken	Spain	CD medium (poor)	61	Low	Ampicillin, amoxicillin	28.7	-	-
599	SA, EASSA 2;P3S329-331	Chicken	Spain	CD medium	388	Low	-	28.7	-	-
606	SA, EASSA 2;P3S395-397	Chicken	Spain	CD medium	792	Medium	-	172.8 (shoulder) and 30.5	-	-
609	SA, EASSA 2;P3S434-436	Chicken	Spain	CD medium	409	Medium	-	29.6	-	-
618	SA, EASSA 2;P3S545-547	Chicken	Spain	CD medium	885	High	S	29.6	Present	1102
710	SA, EASSA 2;A4S13981400K3	Chicken	Spain	CD medium	832	Medium	S	32.6	Present	1095
720	SA, EASSA 2;A4S514516S3	Chicken	Spain	CD medium	714	Medium	S	32.6	-	-
732	SA, EASSA 2;A4S964966S3	Chicken	Spain	CD medium	879	High	Ampicillin	85.7 (shoulder) and 36.5	-	-
910	SA, EASSA 2; 16ex	Bovine	France	CD medium (poor)	205	Low	-	32.6	-	-
963	SA, EASSA 2;4458 ex	Chicken	France	CD medium	577	Medium	-	74.8 (shoulder) and 32.6	-	-
D24359	Malawi-Liverpool-Wellcome Trust Clinical Research Programme	Human, invasive isolate	Malawi	CD medium	1097	High	S	32.6	-	-

EASSA: European Antimicrobial Susceptibility Surveillance in AnimalsCD: Chemically DefinedS: sensitive to ampicillin, amoxicillin, aztreonam, cefotaxime, ceftazidime, ceftriaxone, ciprofloxacin, chloramphenicol, cotrimoxazole, meropenem

*avMW: average molecular weight, as determined by HLP-SEC

** values represent the geometric mean of the fluorescent signal as determined by FACS

#### *S*. Typhimurium isolates

The second selection criterion was based on the production of OAg. OAg extracted from the selected strains were quantified and the isolates were categorized as low (< 400 μg/ml), medium (400–850 μg/ml) and high producing (≥ 850 μg/ml) strains (Table 1).

Nine strains were selected as high producers, but since we wanted to select strains with the highest safety profiles, one isolate was excluded because of resistance to aztreonam (1419), and another because of its human origin (2196). The OAg from the remaining 7 strains (1417, 1418, 1421, 2189, 2192, 2193, and 2198) were analyzed by Size Exclusion-High Pressure Liquid Chromatography (HPLC-SEC), Nuclear Magnetic Resonance (NMR) and flow cytometry.

**Fig 1:**
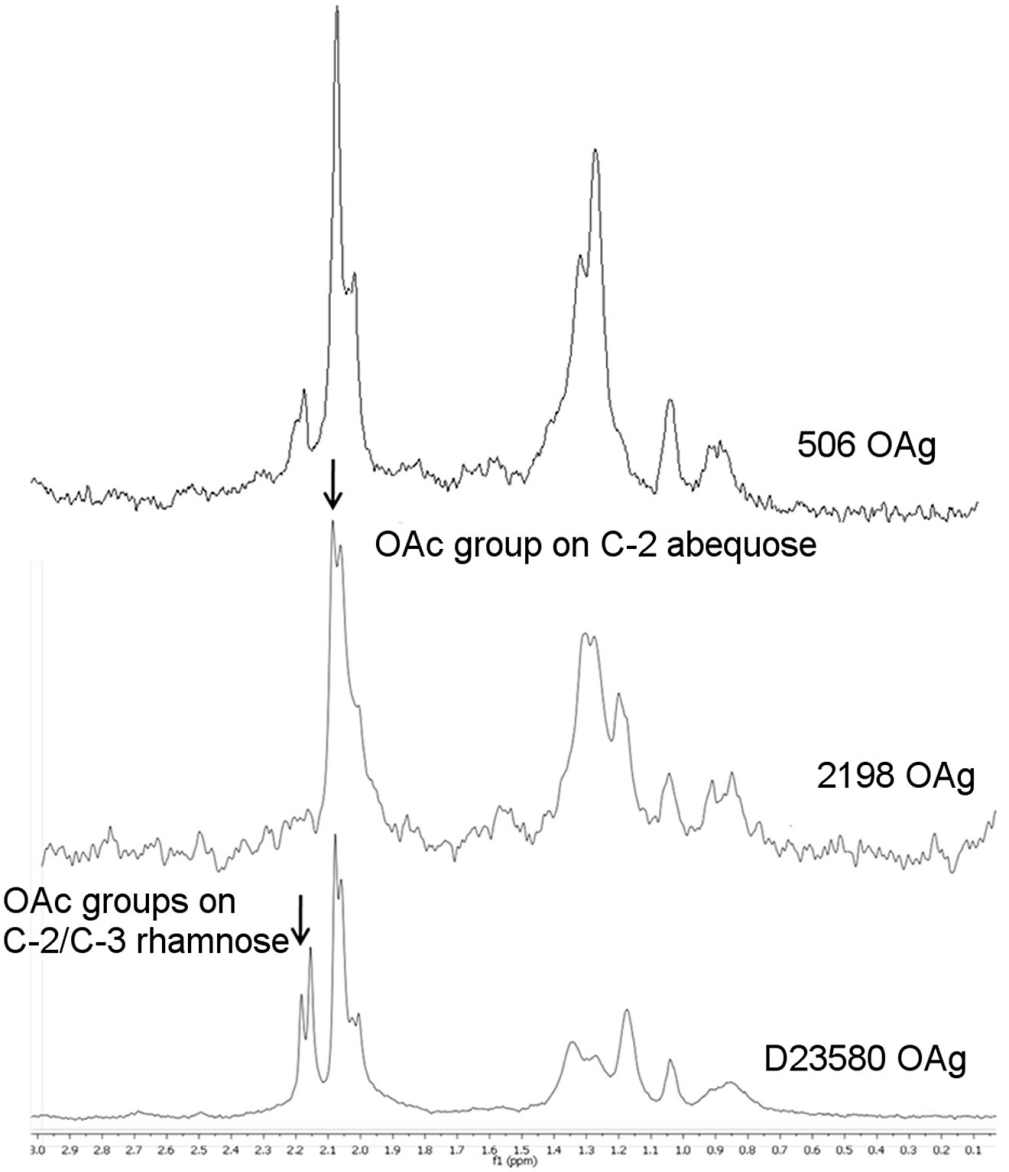
HR-MAS NMR showing O-acetylation patterns for *S*. Typhimurium D23580 and 2198 OAg and *S*. Enteritidis 506 OAg, as examples. O-acetylation sites for *S*. Typhimurium OAg have been determined [30]. Determination of sites for *S*. Enteritidis is ongoing.

OAg profiles showed peaks at high (HMW) or medium (MMW) molecular weight, in the range 63.8–87 kDa and 34.5–28.3 kDa, respectively [29, 30]. One isolate presented only MMW OAg (2192), while five presented both HMW and MMW populations (1417, 1418, 1421, 2189 and 2198) and one isolate (2193) showed only peaks at very low molecular weight (LMW), with very few/no repeating sugar units in the OAg chain (Table 1). Proton High-Resolution Magic Angle Spinning Nuclear Magnetic Resonance (HR–MAS NMR) was performed directly on the bacteria and allowed detection of the typical OAg signals and identification of O-acetylation levels and positions. As well as D23580, one other isolate (1421) was O-acetylated on both rhamnose (Rha) and abequose (Abe), while the remaining isolates were O-acetylated on Abe only (Table 1, Fig 1).

**Fig 2:**
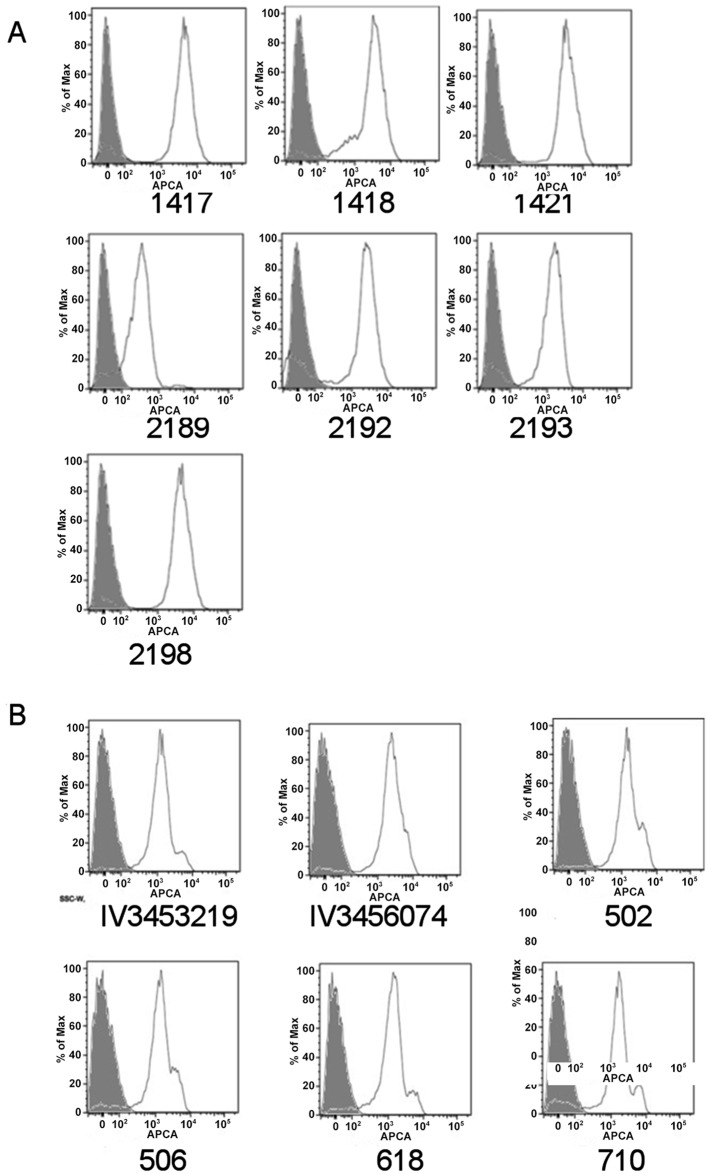
FACS analysis of selected *S*. Typhimurium (A) and *S*. Enteritidis strains (B). Bacteria were incubated with pooled sera of mice immunized with conjugates synthesized using OAg from invasive human strain *S*. Typhimurium D23580 (A) and *S*. Enteritidis D24359 (B).

FACS analysis performed with serum from mice immunized with a conjugate generated using OAg from the endemic D23580 strain [11] showed antibody binding to all 7 *S*. Typhimurium, confirming antigenic similarity between OAg from the invasive African human isolate and the selected animal strains (Fig 2A).

Further selection criteria were applied in order to identify among these 7 strains those most representative of the spectrum of *S*. Typhimurium in terms of OAg structural characteristics. Among the strains with OAg populations at two different average MW and O-acetylation on Abe only (2198, 2189, 1418, 1417), 2189 and 1418 were chosen on the basis of higher OAg production (Table 1). Among the strains presenting one OAg population (2192 and 2193) and O-acetylation only on Abe, 2192 was selected because 2193 produced mainly core sugars and previous work has shown a lack of immunogenicity for such OAg [16, 31]. Finally, strain 1421 was selected as the only representative strain presenting two OAg populations and O-acetylation on both Rha and Abe, similar to the D23580 African isolate.

#### *S*. Enteritidis isolates

The 19 strains selected based on growth characteristics in CD medium, were evaluated for OAg production. One strain was a low producer (< 400 μg/ml). There were 13 medium (400–850 μg/ml) and five high producers (≥ 850 μg/ml). In contrast to the *S*. Typhimurium OAg, less variability in MW distribution was found by HPLC-SEC analysis. All the strains produced a major species of similar average MMW of about 30 kDa (Table 2).

**Fig 3:**
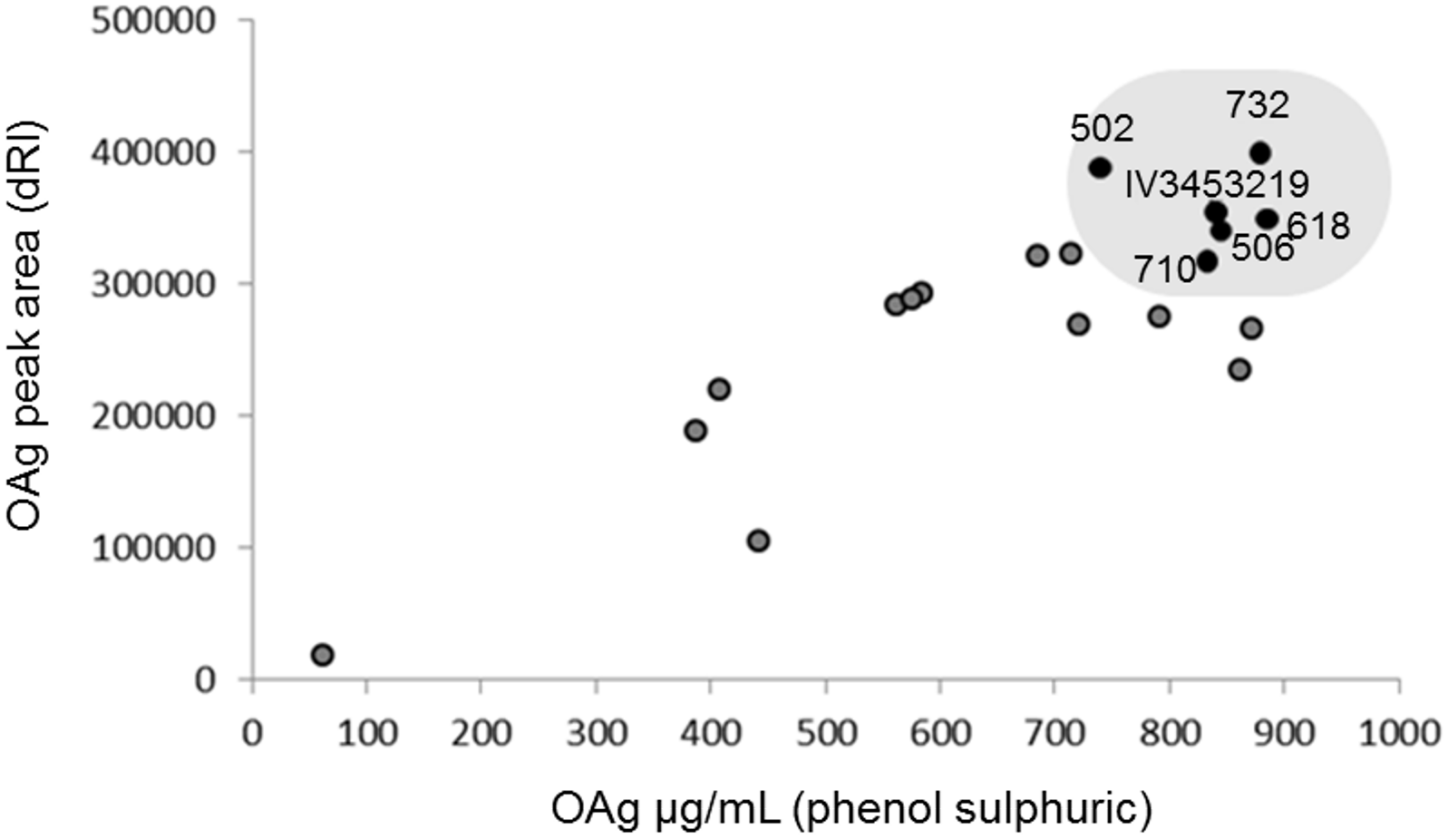
Comparison between *S*. Enteritidis OAg amount and OAg peak area. The OAg total sugar content was calculated by phenol sulfuric assay, and peak area of OAg (allowing exclusion of core sugars with few or no OAg repeating units) by HPLC-SEC. Shaded area indicates the six selected *S*. Enteritidis isolates with highest values of both parameters.

Because of the similarity of OAg MW, in addition to quantification by phenol sulfuric assay, *S*. Enteritidis sugar production was quantified by OAg peak areas, as measured by HPLC-SEC (dRI, differential Refractive Index). While phenol sulfuric assay estimates the total sugar content, including core sugars with few or no OAg repeating units, analysis by HPLC-SEC allows selection of peaks related to longer OAg, normally corresponding to MMW chains, which we targeted for conjugate production [11]. Quantification of longer OAg chains by HPLC-SEC correlated (p<0.05 by Spearman correlation) with total sugar content by phenol sulfuric assay (Fig 3).

From this comparison, 6 isolates (IV3456074, IV3453219, 502, 506, 618 and 710) were selected which showed high sugar production as quantified by both phenol sulfuric assay and HPLC-SEC. Therefore, for these isolates high sugar levels were mostly due to long OAg-chains rather than LMW populations. *Salmonella* FACS analysis, performed with serum containing anti-OAg antibodies raised to the endemic D24359 invasive isolate, confirmed good binding of these antibodies to all isolates (IV3453219, IV3456074, 502, 506, 618, 710) (Fig 2B). *S*. Enteritidis isolates showed less variability than *S*. Typhimurium in terms of O-acetylation, and all were characterized by having low levels (≤ 30%) of O-acetyl groups (Fig 1).

### Fermentation and OAg Purification

#### *S*. Typhimurium isolates

The four selected *S*. Typhimurium strains were fermented in CD medium and high cell densities (OD>35) were reached in less than 15 h (duplication time ranged from 1.2 to 1.6 h) for all, except strain 1421 (S1A Fig), which reached a plateau at OD 7 with no further growth. On the basis of these fermentation findings, 1421 was down-selected and OAg was purified from 2189, 1418 and 2192 strains.

Two liters of fermentation broth were subjected to acid hydrolysis and OAg were further purified from 0.8–1.9 L hydrolyzed broth [29]. The purification process gave good overall yields (always above 70%) and 15.7–18.4 mg/OD of pure OAg were obtained per L of fermentation broth, according to the specific strain. Considering that fermentations were stopped at ODs ranging from 34 to 57, 0.5 to 1.0 g of OAg were recovered per L of fermentation broth.

#### *S*. Enteritidis isolates

The 6 selected *S*. Enteritidis strains were fermented in CD medium, but fermentation was not successful for strains IV3456074, 506 and 710 which reached ODs of 7–10 only (reasons and possible improvements in fermentation were not investigated). In contrast, strains IV3453219, 502 and 618 grew to satisfactory cell densities (S1B Fig). 618 required a more complex medium, but this was still compatible with large-scale production (medium ingredients not derived from animal sources). As for *S*. Typhimurium isolates, OAg purification recoveries for *S*. Enteritidis were > 65% and 12.7–24.2 mg/OD of pure OAg were obtained per L of fermentation broth.

### OAg Characterization

All purified OAg were characterized by good purity, with protein and nucleic acid content <1% (weight to weight with respect to sugar content) and endotoxin level < 0.1 EU/μg of sugar. High-Performance Anion-Exchange pulsed amperometric detection (HPAEC-PAD) and ^1^H NMR analysis confirmed the presence of the expected sugars constituting the OAg chain, with rhamnose, mannose, galactose and abequose (*S*. Typhimurium) or tyvelose (*S*. Enteritidis) in 1:1 molar ratios. All *S*. Enteritidis OAg showed low glucosylation levels (from 9 to 15%), while more variability was found for *S*. Typhimurium OAg (from 24 to 84%). As determined by HR-MAS NMR performed directly on bacteria, O-acetylation levels on purified OAg were low for all *S*. Enteritidis strains and variable for *S*. Typhimurium (Table 3).

**Table 3:** Main characteristics of OAg selected for conjugate production and of the corresponding conjugates.

	Glc (%)	OAc (%)	Kd	Kd unconjugated OAg	OAg/CRM_197_ w/w ratio
*S*. Typhimurium **2192**	24	100	0.49	0.67	1.7
*S*. Typhimurium **1418**	84	73	0.48	0.56 and 0.67	1.7
*S*. Typhimurium **2189**	51	58	0.46	0.57 and 0.68	2.0
*S*. Typhimurium **D23580**	39	142	0.44	0.58 and 0.68	2.1
*S*. Typhimurium **LT2**	11	64	0.47	0.58 and 0.68	1.9
*S*. Typhimurium **SL1344**	8	75	0.43	0.58 and 0.68	2.1
*S*. Enteritidis **502**	12	22.4	0.50	0.62 and 0.53	1.7
*S*. Enteritidis **618**	15	10	0.55	0.62	1.2
*S*. Enteritidis **IV3453219**	11	31.6	0.52	0.65 and 0.52	1.8
*S*. Enteritidis **D24359**	9	35.4	0.52	0.62	1.2

Because of their high variability, OAg from *S*. Typhimurium isolates have been characterized in further detail by Micoli et al. [30].

### OAg Conjugation to CRM_197_

Acid hydrolysis was performed on the bacteria to extract OAg (plus core sugars) by removing lipid A. This process cleaves the labile linkage between the lipid A and the KDO sugar at the end of the core region. This sugar, quantified by semicarbazide/HPLC-SEC method [29] was in a ratio close to 1 with N-acetyl glucosamine (GlcNAc, a unique sugar of the core region, determined by HPAEC-PAD) for all the OAg, confirming that the delipidation process left one KDO sugar per OAg chain. The KDO was used for introducing ADH and then SIDEA linkers, and for binding the OAg to CRM_197_ [11, 32, 33]. For all OAg, conjugation was efficient, with no residual unconjugated protein in the mixture. Purification by hydrophobic interaction chromatography allowed removal of unconjugated sugar chains.

The main characteristics of the conjugates obtained are reported in Table 3. For all of them, Kd values were higher than those of free OAg and free protein (Kd of CRM_197_ of 0.72), confirming the larger size of the conjugate with respect to free components. All conjugates presented a similar saccharide to protein weight/weight ratio.

### Immunogenicity Studies

#### *S*. Typhimurium conjugates

**Fig 4:**
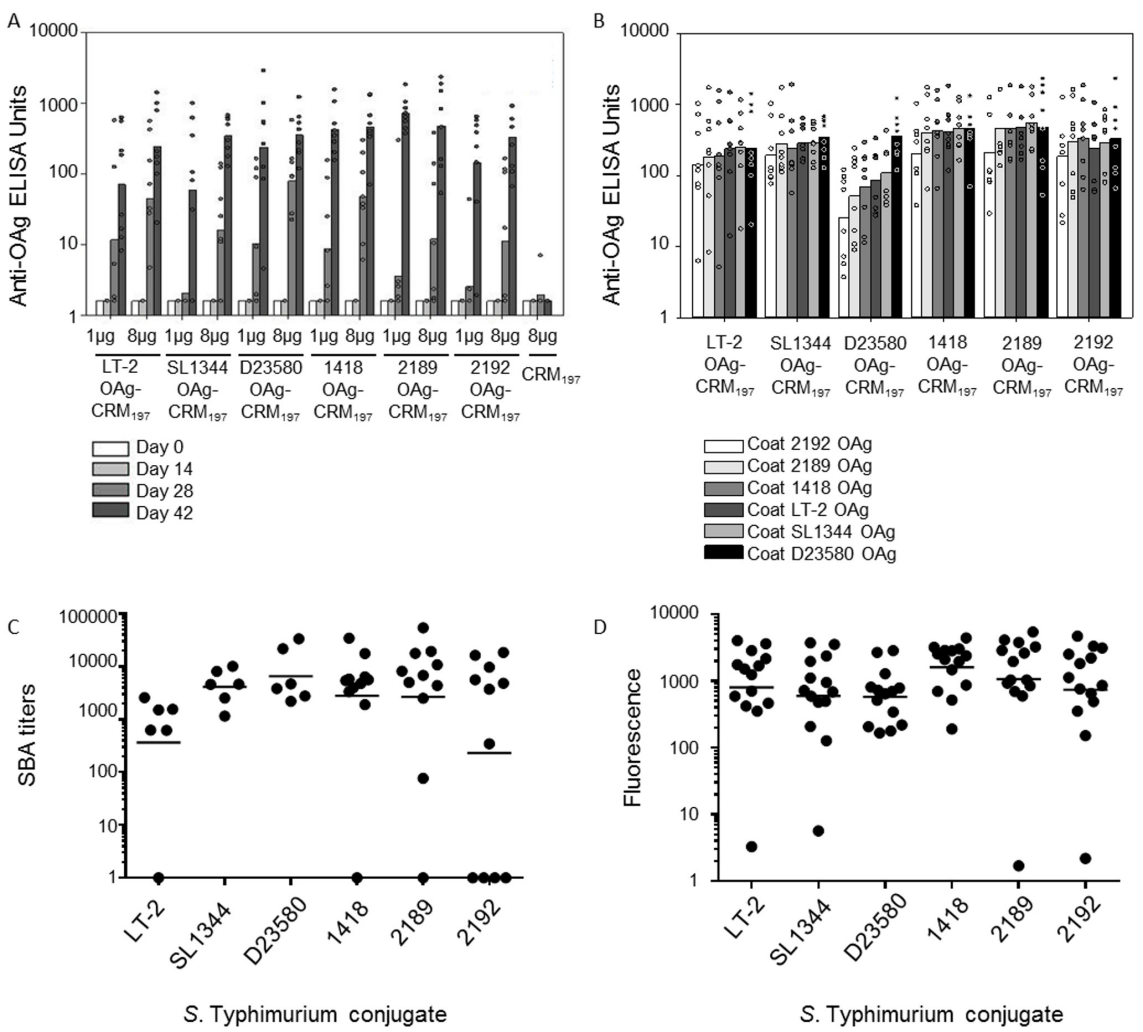
Immunogenicity of *S*. Typhimurium OAg-conjugate vaccines. (A) Anti-OAg IgG at different time-points following immunization with various conjugates administered at 1 and 8 μg doses (D23580 OAg was used as coating agent); (B) Anti-OAg IgG response of the six conjugates using both homologous and heterologous OAg for plate coating (day 42, 8 μg/dose); (C) Serum bactericidal Activity (SBA) performed using pooled sera of mice belonging to the same immunization group against a total of 11 *S*. Typhimurium strains. Not all strains were tested against each conjugate-serum. Lab strains LT2, SL1344; Malawi endemic isolates: D23580, D25352, D22477, D24545, D24533; Kenya endemic isolates: Ke237, Ke238, Ke244, Ke249 (solid bars represent the geometric means; each dot represents a different *S*. Typhimurium isolates); (D) FACS analysis performed using pooled sera of mice belonging to the same immunization group of 14 *S*. Typhimurium strains (lab strains: LT2 and SL1344, animal isolates: 1418, 2189, 2192; Malawi endemic strains: D23580, D22477, D24533, D24545, D25352; Kenyan endemic strains: Ke237, Ke238, Ke244, Ke249) incubated with sera from mice immunized with all *S*. Typhimurium conjugates (solid bars represent the geometric means; each dot represents a different *S*. Typhimurium isolates).

Groups of eight mice were immunized three times at two week intervals with six different *S*. Typhimurium conjugates synthesized using OAg from six different strains (Table 3): 2192, 1418, 2189 are the animal strains selected after the screening process, D23580 is the invasive human isolate, LT2 and SL1344 are common laboratory isolates that were included as additional comparators. All *S*. Typhimurium conjugates were immunogenic showing dose-dependency of the anti-OAg IgG response (Fig 4A). Anti-OAg IgG levels measured by Enzyme Linked Immunosorbent Assay (ELISA) (OAg purified from D23580 was used as coating agent) were not significantly different for the group of *S*. Typhimurium conjugates when tested at 8 μg/dose.

At the 1 μg dose, the 2189 conjugate elicited significantly higher anti-OAg IgG than LT2- and SL1344-conjugates at day 42, (p = 0.04) (Fig 4A). When the same vaccine sera were tested by ELISA against all six OAg (purified and used as ELISA plate coating agents) (Fig 4B), a strain-specific response was clearly detected with the D23580-conjugate, which induced high anti-OAg IgG levels when measured against the homologous D23580 OAg, but lower anti-OAg IgG levels when measured against OAg from all other sources (significant differences, p<0.05, found between anti-D23580 OAg and anti–1418, -2189 and -2192 OAg). No such strain specificity was found for the other sera (Fig 4B). Significantly higher anti-OAg IgG were detected in sera from 1418- and 2189-conjugates, compared with D23580- conjugate, when the ELISA plate coating was with LT2, SL1344 and 2189 OAg.

**Fig 5:**
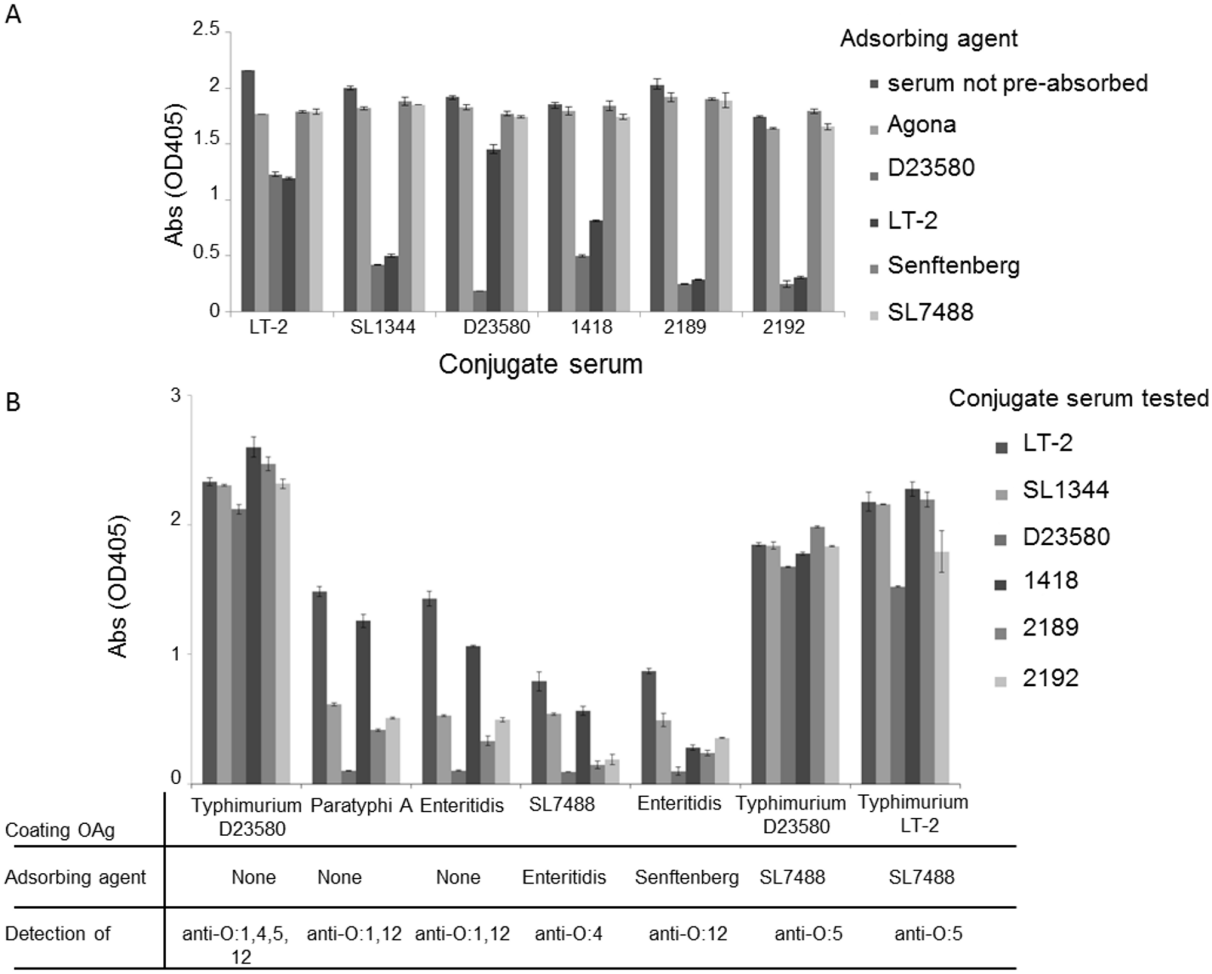
Fine specificities of anti-OAg IgG elicited by the 6 different *S*. Typhimurium conjugates determined by ELISA. (A) Pre-adsorption of mouse conjugate sera with the *Salmonella* serovars/strains in Table 4. *S*. Typhimurium D23580 OAg used as coating agent; (B) Pre-adsorbed and unabsorbed mouse sera with OAg coating from different *Salmonella* serovars/strains. STm: *S*. Typhimurium; SEn: *S*. Enteritidis.

To further understand the specificities of the anti-OAg IgG raised to the vaccines, mouse sera were incubated with different *Salmonella* serovars/strains containing different OAg determinants, so that antibodies recognizing fine specificities present on each strain could be adsorbed out and a signal reduction, attributable to a lack of those antibodies, recorded (Table 4, Fig 5A).

**Table 4:** Fine specificities of anti-OAg antibodies that can be detected in sera of mice immunized with *S*. Typhimurium OAg-conjugates, according to pre-adsorption strain and ELISA coating antigens.

	ELISA OAg coating			
**Pre-adsorption**	*S*. Typhimurium D23580 (O:1,4,5,12; Rha-acetylation)	*S*. Typhimurium LT2 (O:1,4,5,12)	*S*. Paratyphi A (O:1,2,12)	*S*. Enteritidis (O:1,9,12)
None	O:1,4,5,12; Rha-acetylation	O:1,4,5,12	O:1,12	O:1,12
*S*. Enteritidis (O:1,9,12)	NA	NA	O:2 (anti-O:1,12 adsorbed out)	NA
SL7488 (O:1,4,12)	O:5; Rha-acetylation (anti-O:1,4,12 adsorbed out)	O:5 (anti-O:1,4,12 adsorbed out)	NA	NA
*S*. Senftenberg (O:1,3,19)	O:4,5,12; Rha-acetylation (anti-O:1 adsorbed out)	NA	NA	O:12 (anti-O:1 adsorbed out)
*S*. Agona (O:4,12)	O:1,5; Rha-acetylation (anti-O:4,12 adsorbed out)	NA	NA	NA
*S*. Typhimurium LT2 (O:1,4,5,12)	Rha-acetylation (anti-O:1,4,5,12 adsorbed out)	NA	NA	NA
*S*. Typhimurium D23580 (O:1,4,5,12; Rha-acetylation)	Nil (anti-Rha-acetylation and anti-O:1,4,5,12 adsorbed out)	Nil (anti-O:1,4,5,12 adsorbed out)		

Rha: rhamnose. NA: results not available

Anti-OAg ELISA signal was reduced for all conjugate sera when pre-adsorbed with *S*. Typhimurium strains (D23580 and LT2), consistent with most anti-OAg IgG targeting O:4,5. Adsorption with the other serovars did not produce a substantial decrease in ELISA signal, suggesting that either relatively little anti-O:1 and O:12 IgG are generated, or they bind OAg poorly compared to anti-O:4,5 IgG. D23580-conjugate serum demonstrated strain-specificity once again, with strong ELISA signal reduction occurring only when the serum was incubated with homologous D23580 strain, but not with LT2. This suggests that anti-D23580 OAg IgG are mainly directed against a specific D23580 epitope not present on other *S*. Typhimurium OAg. We speculate that this epitope is formed by the additional O-acetylation groups detected on the Rha residue of the OAg repeating unit (Table 4, Fig 5A) [11, 30]. Finally, we searched for anti-OAg IgG directed against internal determinants, such as O:1,12, by coating the ELISA plates with OAg from different *Salmonella* serovars/strains and incubating conjugate sera either not adsorbed, or following specific adsorption (Table 4, Fig 5B). These experiments suggested that the majority of anti-OAg IgG elicited by all conjugates were against the O:5 determinant, as shown by testing sera pre-adsorbed with SL7488 (a strain expressing O:1,4,12, so that anti-O:1,4,12 IgG would be adsorbed out) against OAg from both *S*. Typhimurium D23580 and LT2 (a situation in which remaining anti-O:5 IgG could be detected). Anti-O:4, anti-O:1 and anti-O:12 IgG were also detected, but at much lower levels than anti-O:5 IgG. The only exception was D23580-conjugate, which did not appear to elicit IgG with anti-OAg specificities other than to O:5 and Rha-acetylation. When evaluating ELISA signals to O:1,12 (coating with *S*. Paratyphi A or *S*. Enteritidis), the LT2 and 1418-conjugates elicited more specific antibodies than the other conjugates. Comparing this O:1,12 signal with O:12 only (coating with *S*. Enteritidis and pre-adsorption of sera with *S*. Senftenberg), there was a strong signal reduction with O:12 alone when testing serum raised to the 1418-conjugate, but not the LT2 conjugate. The finding suggests that more anti-O:1 than anti-O:12 IgG is induced by the 1418 vaccine, and the opposite for the LT2 vaccine. This is consistent with the known OAg structure of 1418 which is characterized by glucosylation of galactose at C6 [30], the basis of O:1 determinant.

Analysis of serum bactericidal activity (SBA) showed that antibodies induced by *S*. Typhimurium conjugates induced different levels of susceptibility/resistance to killing (Fig 4C). Of a total of 11 strains tested (not all strains were tested against each conjugate, more details in S1 Table), 1 Malawi strain (D25352) was completely resistant to killing when tested with the three selected *S*. Typhimurium conjugate. The 2192 conjugate was unable to kill 4 of the 11 strains. By FACS analysis (Fig 4D, S2 Table), all sera from the *S*. Typhimurium conjugates were able to bind 13 out of 14 *S*. Typhimurium strains tested, with one Malawi isolate (D24533) bound only by sera from D23580 and 1418 conjugates.

#### *S*. Enteritidis conjugates

**Fig 6:**
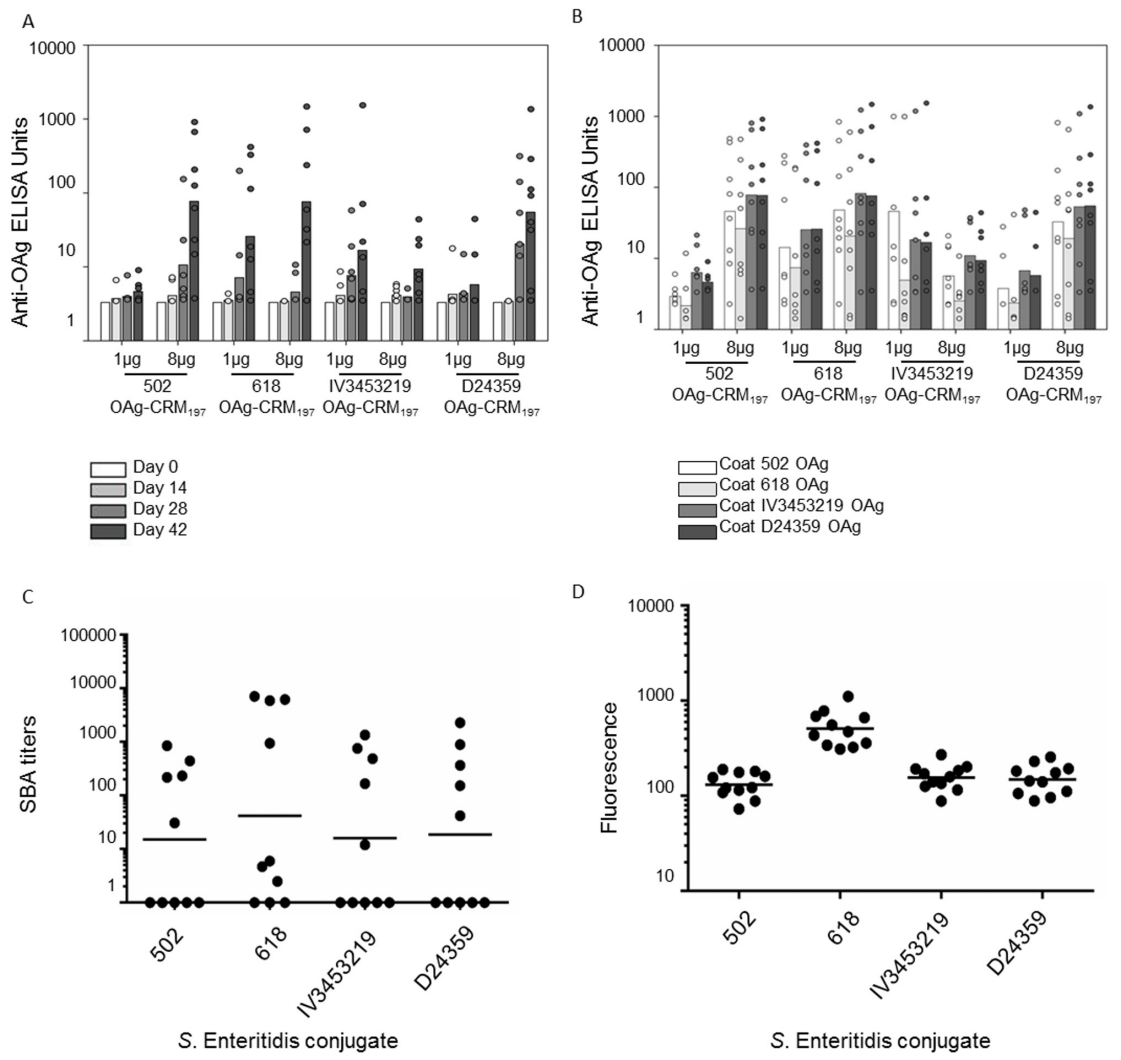
Immunogenicity study with *S*. Enteritidis OAg-conjugate vaccines. (A) Anti-OAg IgG dose-response at different time-points for *S*. Enteritidis conjugates given three times at two weekly intervals with 1 and 8 μg doses (D24359 OAg used as coating agent); (B) response of *S*. Enteritidis conjugates against homologous and heterologous OAg for plate coating (day 42). (C) SBA performed using pooled sera of mice belonging to the same immunization group against 10 *S*. Enteritidis strains (lab isolates: CMCC4314; Malawi endemic isolates: D24359, D24953; Kenya endemic isolates: Ke016, Ke117, Ke180, Ke151; animal isolates: 618, 502, IV3453219) (solid bars represent the geometric means, each dot represents a different *S*. Enteritidis isolates); (D) FACS analysis performed using pooled sera of mice belonging to the same immunization group of 10 *S*. Enteritidis strains (lab isolate: CMCC4314; animal isolates: IV3453219, 502, 618; Malawi endemic strains: D24953, D24359; Kenyan endemic strains: Ke151, Ke180, Ke016, Ke117) incubated with sera from mice immunized with all *S*. Enteritidis conjugates (solid bars represent the geometric means, each dot represents a different *S*. Enteritidis isolates).

A parallel immunogenicity study, with three immunizations of 1 μg or 8 μg at two weeks intervals in groups of eight mice, was conducted to evaluate the four *S*. Enteritidis conjugates synthesized using OAg from 4 different strains (Table 3): 502, 618, IV3453219 were the animal strains selected from the screening process, while D24359 is the invasive human isolate included as comparator. Low anti-OAg IgG were induced with the 1 μg dose (Fig 6A), while higher IgG levels were present following immunization with the 8 μg dose, except with the IV3453219-conjugate.

There was large intergroup variation in the antibody responses to the *S*. Enteritidis conjugate vaccines, with more than one non-responding mouse in each group. No clear strain-specific response was detected, consistent with the lack of difference between the OAg extracted from all the *S*. Enteritidis source strains (Fig 6B, Table 3).

SBA suggested that *S*. Enteritidis strains (10 strains tested, also see S3 Table for details) are generally more resistant to antibody-dependent complement-mediated killing than *S*. Typhimurium strains. Geometric mean SBA titers were almost 2 log_10_ lower for *S*. Enteritidis compared with *S*. Typhimurium, and some isolates were fully resistant to killing. Nevertheless, all *S*. Enteritidis conjugate vaccines elicited sera with bactericidal activity against some *S*. Enteritidis isolates (Malawi strains D24359 and D24953 and two animal isolates 618 and 502 were completely resistant to complement-mediated killing) and no significant differences were detected between the killing abilities of the different sera (Fig 6C).

By FACS analysis, antibodies raised to all *S*. Enteritidis conjugates were able to bind all the 10 strains tested, with 618-conjugate sera giving the highest fluorescence values (Fig 6D, S4 Table).

## Discussion

With the final aim of producing an OAg-based vaccine against *S*. Typhimurium and Enteritidis, we investigated a panel of NTS isolates to identify the most suitable OAg sources for OAg-CRM_197_ glycoconjugate vaccine development. An overview of the selection process we underwent is given in Table 5.

**Table 5:** Workflow of strain selection process.

Selection Criteria	Outcome for *S*. Typhimurium	Outcome for *S*. Enteritidis
Growth in CD medium	• 30 Typhimurium isolates screened • 27 selected	•21 Enteritidis isolates screened •19 selected
OAg production level (>850 μg/ml)	• 9 selected for next step ○ 1417, 1418, 1421, 2189, 2192, 2193, 2198, 1419 and 2196	•6 selected for next step ○ IV3456074, 506, 710, IV3453219, 502 and 618
Safety (exclusion of isolates with antibiotic resistance and human origin)	•1419, 2196 excluded •7 selected for next step ○ 1417, 1418, 1421, 2189, 2192, 2193, and 2198	•No exclusion •Same 6 selected for next step
FACS analysis with serum from mice immunized against invasive Typhimurium/Enteritidis OAg	•No exclusion •Same 7 selected for next step	•No exclusion •Same 6 selected for next step
OAg populations and O-acetylation levels representative for the spectrum of OAg features	•3 excluded •4 selected ○ 1418, 2189 (OAg populations at two MW, O-acetylation on Abe) ○ 2192 (OAg populations at 1 MW, O-acetylation on Abe) ○ 1421 (OAg populations at 2 MW, O-acetylation on both Rha and Abe)	•No exclusion •Same 6 selected for next step
Fermentation	•1421 excluded •3 selected for immunogenicity studies ○ 1418, 2189 and 2192	•3 excluded: IV3456074, 506 and 710 •3 selected for immunogenicity studies ○ IV3453219, 502 and 618

CD: Chemically Defined

The majority of *S*. Typhimurium and *S*. Enteritidis strains in our collections were able to grow in defined medium and produced a wide range of OAg quantities. Fermentation of selected high OAg-producing NTS strains (non-invasive and antibiotic susceptible) was effective and yielded up to 24.2 mg/OD/L. This amount could translate into up to 40 g of purified OAg, obtained from one single 50 L fermentation run, considering a final OD 35 and an average purification process yield of 70%. By way of comparison, this corresponds to more than 4 times the amount of Vi antigen that is recovered from *Salmonella* Ty2 in a production process [34]. These evaluations are particularly important for vaccines needed in resource-poor settings, where developing country manufacturers may perform cGMP vaccine production and low costs and feasibility of manufacture are crucial elements.

Two of the three *S*. Typhimurium strains that were selected as OAg source (1418 and 2189), produced OAg of similar molecular weight, with two main populations of 28–31 and 80–87 kDa [30]. One isolate (2192) presented only one main peak of approximately 34.5 kDa [30]. Glucosylation levels ranged from 24% (2192) to 84% (1418), and O-acetylation levels (on Abe) from 58% (2189) to 100% (2192) (Table 3). Although not part of the selection process, we also examined two laboratory isolates and the Malawian D23580 invasive isolate. Their OAg presented similar features to those of the animal isolates, except for the additional O-acetylation site on Rha found in the Malawian isolate [30] (Table 3). Contrary to what has been previously described [35, 36], the *S*. Enteritidis isolates tested were more homogeneous than the *S*. Typhimurium isolates, with the majority of them producing only one OAg molecular weight population of 29.6–32.6 kDa with generally low glucosylation and low O-acetylation levels and the same O-acetylation position [37] (Table 3).

The conjugation process employed here does not modify the OAg repeating unit, maintaining native OAg structures, so that immunogenicity could be correlated with OAg structure and specific strain characteristics [33]. Therefore, by preparing conjugates with OAg representative of different structures, we aimed to evaluate the influence of OAg structure on immunogenicity. This was particularly relevant for the *S*. Typhimurium serovar, as it showed the highest OAg diversity. Data obtained with the *S*. Typhimurium conjugates indicated that the presence of Rha-O-acetylation in *S*. Typhimurium D23580 generates an immunodominant epitope with much of the induced anti-OAg antibodies directed against it [11]. The lack of binding, by ELISA (Fig 5B) and by FACS (data not shown), against *S*. Paratyphi OAg, which has also been found O-acetylated on C–2/C–3 Rha [38] (total OAc 68%), may hint for the presence of a structural epitope, specific for *S*. Typhimurium OAg chain. A similarly specific antibody response was not induced by the OAg used to generate the other vaccines. In contrast to the D23580-conjugate, all other OAg-conjugates lacking Rha-O-acetylation induced an antibody response against other OAg determinants, principally O:4 and O:5. When aiming to develop a vaccine with the broadest possible coverage, the strain-specific response identified with the D23580-conjugate is a potential issue, as analysis of OAg structures of endemic Kenyan isolates [37] has shown that invasive isolates may or may not have Rha-O-acetylation. Therefore, a *S*. Typhimurium OAg-based vaccine for Africa would need to induce antibodies capable of binding to both forms of OAg (with or without Rha-O-acetylation). From our findings, the OAg production strain should lack Rha-O-acetylation (as for all three selected Typhimurium strains).

Evaluating whether SBA could discriminate further among Typhimurium conjugates and most specifically between the three selected strains (which were tested against a panel of 11 strains), we found that the 2192-conjugate did not elicit bactericidal antibodies against 4 of 11 isolates. We do not understand the reason for this finding, but *S*. Typhimurium 2192 was the only isolate with a single OAg population and had the lowest glucosylation levels, factors which may both influence the generation of functional anti-OAg antibodies, as suggested in previous work [11]. Although not bactericidal, 2192-conjugate antibodies bound the tested *S*. Typhimurium isolates as for the other conjugates. Nevertheless, isolate D24533 was not bound by IgG elicited by the vaccine sera except for D23580- and 1418-conjugates (S3 Table). Surprisingly, the same isolate was killed in SBA (S1 Table), suggesting potential bactericidal activity of other Ig antibody classes (most likely IgM). Taken together, these data show that ELISA, SBA and FACS studies can provide different insights into the overall immunogenicity of candidate vaccines.

When we examined *S*. Enteritidis conjugates, we found that all produced lower serum titers and antibody-binding fluorescence signals by SBA and FACS. This is consistent with what we and others have previously observed [37, 39], that *S*. Enteritidis isolates are inherently more resistant than *S*. Typhimurium to antibody-mediated killing. We have previously hypothesized that differences in OAg expression levels [37] may partly explain this. Although we have been able to correlate OAg levels with serum resistance for Kenyan *S*. Typhimurium isolates, we were unable to find such correlation for *S*. Enteritidis isolates, indicating that other factors are relevant for this inherent resistance [37].

Consistent with the OAg homogeneity found among *S*. Enteritidis isolates, no isolate-specific antibody responses were identified following immunization with the *S*. Enteritidis conjugates. However, the IV3453219-conjugate generated the lowest anti-OAg IgG levels, and the 618-conjugate generated the highest SBA titers and FACS signal. The reasons for such differences remain unknown, and we speculate that additional factors, beyond what we assessed here, may contribute to differences in magnitude and functionality of the antibody responses elicited by these *S*. Enteritidis conjugates.

In summary, we undertook an extensive strain selection process to identify *S*. Typhimurium and *S*. Enteritidis isolates that could be used as safe and effective source for efficacious vaccines against iNTS disease in Africa. We were able to identify three animal isolates for each serovar with characteristics suitable for large-scale OAg manufacture. The immunogenicity of the corresponding conjugate vaccines was used as a final step for OAg strain selection. Although we were not able to fully explain our findings, we concluded that *S*. Typhimurium strains 1418 and 2189, whose conjugates showed the broadest SBA activity could be selected as promising strains for a *S*. Typhimurium vaccine, while *S*. Enteritidis strains 502 and 618, whose conjugates had the highest immunogenicity, could be selected for a *S*. Enteritidis conjugate vaccine. Considering the ability of adjuvants to influence immunogenicity and breadth of antibody responses, their use is currently being explored for both *S*. Typhimurium and Enteritidis OAg conjugates. A similar approach could be used to determine which strains to use for the development of glycoconjugate vaccine against other bacterial pathogens.

## Materials and Methods

### Origin and Growth of Bacterial Strains

We received 30 *S*. Typhimurium strains from University of Calgary, Canada (*Salmonella* Genetic Stock Centre, SGSC): 20 of them belonged to the *Salmonella* reference collection A (SARA) [40]; 8 belonged to the LT2-collection [41]; 2 were from the lab of Foodborne Zoonoses, Health Canada in 2001 (Table 1). We also obtained 21 *S*. Enteritidis strains from Quotient Bioresearch Limited, UK, which were isolated by the European Antimicrobial Susceptibility Surveillance in Animals (EASSA), coordinated by the European Animal Health Study Centre, Brussels (CEESA). Those strains belonged to CEESA EASSA collections II and III [42] (Table 2). The clinical *S*. Typhimurium (D22477, D24533, D24545, D25352, D23580) and *S*. Enteritidis (D24953, D24359) Malawi isolates were obtained from the Malawi-Liverpool-Wellcome Trust Clinical Research Programme, Blantyre, Malawi. D23580 is a representative invasive Malawian isolate belonging to ST313 sequence type isolated from a bacteraemic child [43, 44]. The clinical *S*. Typhimurium (Ke237, Ke238, Ke244, Ke249) and *S*. Enteritidis (Ke151, Ke180, Ke016, Ke117) Kenyan isolates were obtained from the Kenyan Medical Research Institute (KEMRI) in Nairobi, Kenya [37]. *S*. Typhimurium SL1344 and *S*. Enteritidis CMCC4314 are laboratory isolates [29, 45].

*S*. Agona 20071186 and *S*. Senftenberg 20050439 were obtained from Imperial College London; SL7488 from the University of Cambridge and *S*. Paratyphi A NVGH308 is a clinical isolate obtained from the Oxford University Clinical Research Unit-Nepal (OCRU-Nepal). Isolates were grown in Chemically Defined medium (CD medium) [11, 29, 46]. Glycerol was used as the carbon source with additional casamino acid supplementation (10g/L, BD Biosciences) depending upon the strain.

### Strain Screening and Selection

#### OAg extraction and quantification

Bacteria were grown overnight in 100 mL liquid cultures. Liquid cultures were centrifuged in order to isolate the pellet. Bacterial pellets were washed with PBS and resuspended in water 2% acetic acid (pH 4) at OD 35, before performing acid hydrolysis at 100°C for 3 h. After hydrolysis, supernatant containing OAg were collected by centrifugation, neutralized with 14% NH_4_OH and desalted on a HiTrap desalting 5 mL column, prepacked with Sephadex G–25 Superfine (GE Healthcare). Total sugar content was quantified by phenol sulfuric assay [29, 47].

#### HPLC–SEC analysis

OAg molecular weight (MW) distribution was evaluated by HPLC-SEC as previously described [30, 48]. Samples were run, without pretreatment, on a TSK gel G3000 PWXL column (30 cm x 7.8 mm; particle size 7 μm; cod. 808021) with a TSK gel PWXL guard column (4.0 cm x 6.0 mm; particle size 12 μm; cod. 808033) (Tosoh Bioscience). The mobile phase was 0.1 M NaCl, 0.1 M NaH_2_PO_4_, 5% CH_3_CN, pH 7.2, at the flow rate of 0.5 mL/min (isocratic method for 30 min). Void and bed volume calibration was performed with λ-DNA (λ-DNA MW Marker III 0.12–21.2 kb; Roche) and sodium azide (NaN_3_; Merck), respectively. OAg peaks were detected by differential refractive index (dRI). OAg average MW was estimated on standard dextrans (Sigma) calibration curve.

#### HR–MAS NMR spectroscopy

OAg were characterized directly on the bacteria without performing sample purification as previously described [29]. This method allowed detection of characteristic OAg sugars and O-acetylation pattern.

#### Flow cytometry

Bacteria were grown overnight in CD medium, diluted to OD 0.17 and incubated in 1:200 diluted (PBS, 1% BSA) serum samples obtained from immunized mice [10, 11]. After washing with PBS, bacteria were incubated with secondary anti-mouse APC-labeled antibody (Invitrogen, 1:400 in PBS 1% BSA), fixed in 4% formaldehyde and read using a FACS CANTO (BD Biosciences) flow cytometer (10,000 events recorded). As a negative control, bacteria were incubated with secondary antibody only. Values were obtained by the geometric means of fluorescent signals of positive bacteria (excitation: 650 nm, emission: 668 nm).

### Fermentation of Selected Strains, OAg Purification and Characterization

Bacteria were grown in a 7-L bioreactor (EZ-Control; Applikon) to OD of approximately 35 as previously described [11, 46]. Growth conditions were controlled, keeping the temperature at 37 ± 0.5°C, pH at 6.7 (by addition of NH_4_OH 28%), and pO_2_ saturation at 30% (by agitation and pressure controls in cascade). All NTS strains were fermented in CD medium, except for *S*. Enteritidis 618, which was grown in a complex medium containing: 10 g/L Soytone (BD Bioscience), 5g/L ultrafiltered yeast extract (Difco), 10 g/L NaCl, 3% glycerol. OAg purification was performed as previously described [29]. OAg purity was assessed by micro BCA for protein content, absorption at 260 nm for nucleic acid content and LAL for endotoxin level. MW distribution was evaluated by HPLC-SEC (dRI) and sugar composition by HPAEC-PAD. ^1^H-NMR was used for verifying sugar identity and measure O-acetylation level. KDO was quantified by HPLC-SEC/semicarbazide assay [29, 30].

### Synthesis and Characterization of Conjugate Vaccines

Adipic acid dihydrazide (ADH) and then adipic acid bis(Nhydroxysuccinimmide) (SIDEA) linkers were introduced on the KDO sugar at the end of the core region with slight modifications at the methods previously described [32, 33]. For all *S*. Typhimurium OAg-ADH-SIDEA, purification was performed precipitating the sugar by addition of dioxane (90% volume in the resulting solution), and then washing the pellet with the same organic solvent (ten times with 1/3 of the volume added for the precipitation) before lyophilization. For all *S*. Enteritidis OAg-ADH-SIDEA, the reaction mixture was added to a volume (equal to two times the reaction mixture volume) of HCl 82.5 ppm and mixed at 4°C for 30 min. Under these conditions, unreacted SIDEA precipitated and was separated by centrifugation. OAg-ADH-SIDEA was recovered from the supernatant by precipitation with EtOH-HCl 55 ppm (85% volume in the final mixture). The pellet was washed four times with EtOH (2 volumes with respect to the reaction mixture volume) and lyophilized.

OAg-CRM_197_ conjugates were synthesized as previously described [32, 33]. Conjugates were purified by hydrophobic interaction chromatography on a Phenyl HP column, loading 500 μg of protein for mL of resin in 50 mM NaH_2_PO_4_ 3M NaCl pH 7.2. The purified conjugate was eluted in water and the collected fractions were dialyzed against 10 mM NaH_2_PO_4_ pH 7.2.

The intermediates of conjugation were characterized as previously described [33]. Purified conjugates were characterized by phenol sulfuric acid assay [47] for total sugar content, micro BCA for total protein content and HPLC-SEC (TSK gel 6000-5000PW in series) for verifying conjugate formation in comparison to free protein and to estimate conjugate MW distribution [33].

### Immunogenicity Studies

Two studies were conducted to compare the immunogenicity of *S*. Typhimurium and *S*. Enteritidis glycoconjugates. In both studies, five week-old female C57BL/6 mice (8 mice per group) from Charles River Laboratories were injected subcutaneously three times, at 2 week intervals, with 1 and 8 μg/dose of OAg (200 μL/dose). A control group of mice immunized with CRM_197_ only at 8 μg/dose was included in the first study. Mouse sera were collected before the first immunization (day 0), on immunization days 14 and 28 and again two weeks after the third immunization, on day 42.

### Serum Antibody Analysis by ELISA

Serum IgG levels against OAg were measured by ELISA [10, 28, 33]. Purified OAg from *S*. Typhimurium D23580, SL1344, LT2, 1418, 2189, 2192 and *S*. Enteritidis D24359, 502, 618, IV3453219 were used for ELISA plate coating (5 μg/mL). Mouse sera were diluted 1:200 in PBS containing 0.05% Tween 20 and 0.1% BSA. ELISA units were expressed relative to mouse anti-OAg D23580 [10] or D24359 IgG standard serum curves, with best 4 parameter fit determined by modified Hill Plot. One ELISA unit was defined as the reciprocal of the standard serum dilution that gives an absorbance value equal to 1 in the assay. Each mouse serum was run in triplicate. Data are presented as scatter plots of individual mouse ELISA units, and geometric mean of each group.

#### Adsorption experiments

Pooled sera from each group of mice immunized with 8 μg/dose *S*. Typhimurium conjugates (day 42 sera) were pre-adsorbed with the following *Salmonella* serovars/strains to analyze IgG specificities: *S*. Agona 20071186 (expressing O:4 and O:12), *S*. Typhimurium D23580 (expressing O:1, O:4, O:5 and O:12), *S*. Typhimurium LT2 (expressing O:1, O:4, O:5 and O:12), *S*. Senftenberg 20050439 (expressing O:1, O:3 and O:19) [49], SL7488 (a mutant Thirsk isolate expressing O:4 instead of O:9, in addition to O:1 and 12) [50]. Bacteria were grown and normalized to OD 2 in 5 mL cultures. 750 μL of bacterial cultures were pelleted and washed with PBS and then resuspended in 250 μL of 1:200 diluted sera (in PBS–1% BSA) for overnight incubation at 4μC. Before performing the ELISA with mouse sera, bacteria with specific antibodies bound to their cell surface were removed by centrifugation.

To further dissect anti-OAg specificities, ELISA were also performed on pooled sera from each group of mice immunized with *S*. Typhimurium conjugates by coating plates with 5 μg/ml of purified OAg from: 1. *S*. Typhimurium D23580 (to test non pre-adsorbed sera, and sera after pre-adsorption with SL7488); 2. *S*. Paratyphi A (NVGH308, expressing O:1, O:2 and O:12); 3. *S*. Enteritidis D24359, expressing O:1, 9, 12 (to test non-pre-adsorbed sera, and sera after pre-adsorption with *S*. Senftenberg); 4. SL7488 (to test sera pre-adsorbed with *S*. Senftenberg); 5. *S*. Typhimurium LT2 (to test sera pre-adsorbed with SL7488) (Table 4).

### SBA

Equal volumes of day 42 mouse serum, belonging to the same group of mice immunized with 8 μg/dose conjugates, were pooled for SBA experiments. *S*. Typhimurium and *S*. Enteritidis bacteria were grown in Luria Bertani (LB) medium to log-phase (OD: 0.2), diluted 1:15,000 in SBA buffer (50 mM phosphate; 0.041% MgCl_2_ 6H_2_O; 33 mg/mL CaCl_2_; 0.5% BSA) and distributed into sterile polystyrene U bottom 96-well microtiter plates (15 μL/well). To each well (final volume 150 μL, 500–1000 colony forming units (CFU)/mL depending on the strain), serum samples serially diluted 5-fold (starting from 1:100 in well dilution) were added. Sera were heated at 56°C for 30 min to inactivate endogenous complement. Active Baby Rabbit Complement (BRC, Cederlane CL3441 lot6288) used at 50% of the final volume was added to each well. BRC source, lot and percentage used in the SBA reaction mixture were previously selected for low toxicity against *S*. Typhimurium and *S*. Enteritidis strains. To evaluate possible nonspecific inhibitory effects of BRC or mouse serum, bacteria were also incubated with the same tested sera plus heat-inactivated BRC; sera alone (no BRC); SBA buffer and active BRC. Each sample and control was tested in triplicate. One hundred microliter reaction mixture from each well was spotted on LB-agar plates at time zero (T0) to assess initial CFU, and at 3h (T180) after incubation at 37°C. LB-agar plates were incubated overnight at 37°C and resulting CFU were counted the following day. Bactericidal activity was determined as serum dilutions necessary to obtain 50% percent CFU reduction at T180 compared with T0. Serum titers equal to 1 were given when no bactericidal activity was detected.

### Statistical Analysis

Statistical analysis of ELISA results was conducted on day 42 samples (mice receiving 8 μg/dose). Groups were compared using Kruskal-Wallis One-Way ANOVA. Post hoc analysis was performed using Student-Newman-Keuls test (using α = 0.05).

### Ethical Statement

The mouse immunization experiments performed at the Novartis Animal Facility in Siena, Italy, complied with the relevant guidelines of Italy (Italian Legislative Decree n. 116/1992) and the institutional policies of Novartis. The animal protocol was approved by the Animal Welfare Body of Novartis Vaccines, Siena, Italy, and by the Italian Ministry of Health (Approval number AEC201018).

## Supporting Information

S1 Fig Representative fermentations of the selected nontyphoidal *Salmonella* strains. (A) *S*. Typhimurium, (B) *S*. Enteritidis. Td: duplication time.
(TIF)

S1 Table SBA values obtained testing pooled sera from mice immunized with *S*. Typhimurium conjugates. Bactericidal activity was determined as serum dilutions necessary to obtain 50% percent CFU reduction at T180 compared with T0. Serum titers equal to 1 were given when no bactericidal activity was detected.
(DOCX)

S2 Table FACS values obtained testing pooled sera from mice immunized with *S*. Typhimurium conjugates. (DOCX)

S3 Table SBA values obtained testing pooled sera from mice immunized with *S*. Enteritidis conjugates. Bactericidal activity was determined as serum dilutions necessary to obtain 50% percent CFU reduction at T180 compared with T0. Serum titers equal to 1 were given when no bactericidal activity was detected.
(DOCX)

S4 Table FACS values obtained testing pooled sera from mice immunized with *S*. Enteritidis conjugates. (DOCX)
