# Supplementary figures and images for: Strain Selection for Generation of O-Antigen-Based Glycoconjugate Vaccines against Invasive Nontyphoidal Salmonella Disease
Source: PLoS One. 2015 Oct 7;10(10):e0139847. doi: 10.1371/journal.pone.0139847 (PMC4596569; doi:10.1371/journal.pone.0139847)

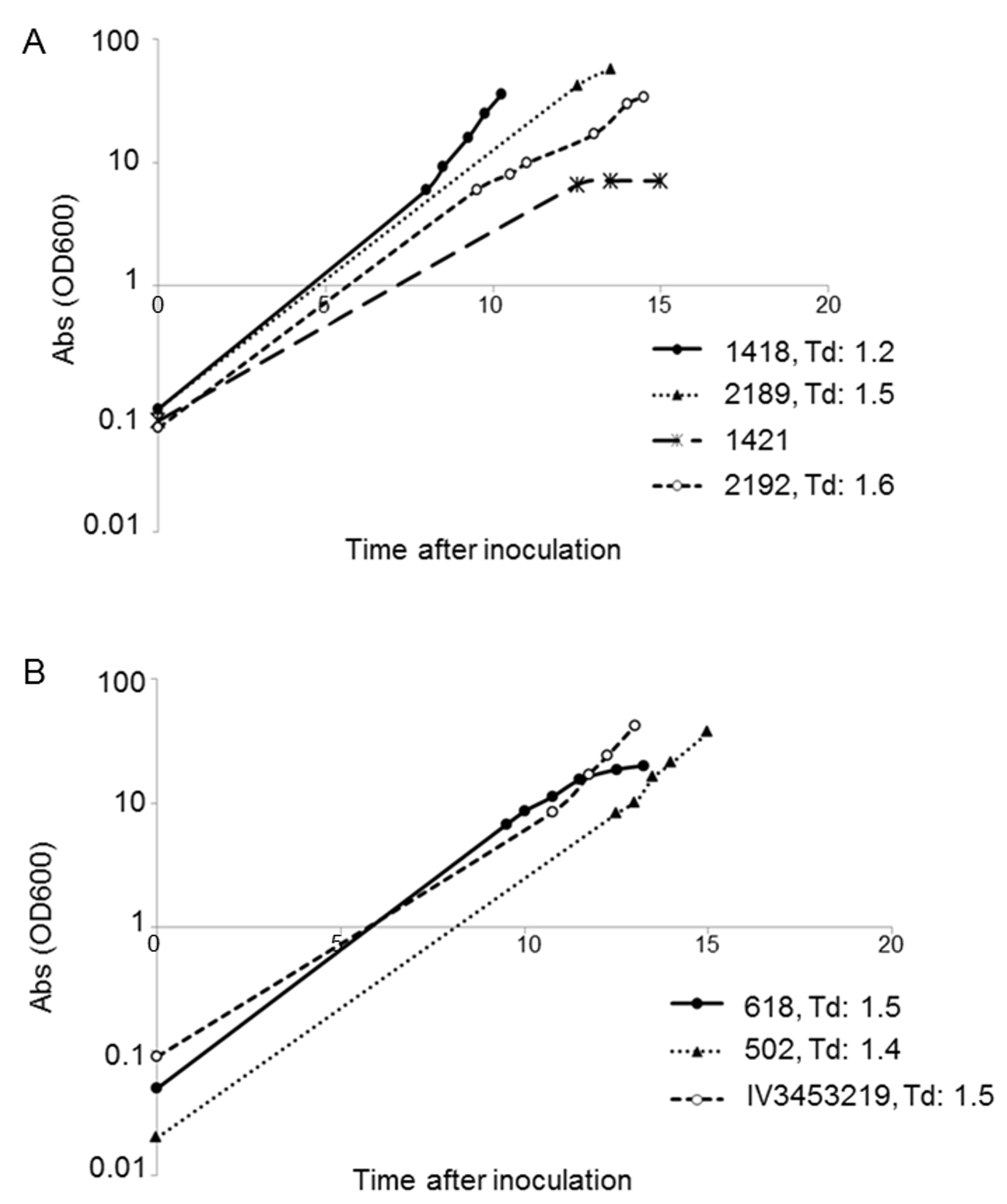

Supplement: S1 Fig — (A) S. Typhimurium, (B) S. Enteritidis. Td: duplication time. (TIF) [file pone.0139847.s001.tif]
