# Supplementary material for: Strain Selection for Generation of O-Antigen-Based Glycoconjugate Vaccines against Invasive Nontyphoidal Salmonella Disease
Source: PLoS One. 2015 Oct 7;10(10):e0139847. doi: 10.1371/journal.pone.0139847 (PMC4596569; doi:10.1371/journal.pone.0139847)
Supplement: S1 Table — Bactericidal activity was determined as serum dilutions necessary to obtain 50% percent CFU reduction at T180 compared with T0. Serum titers equal to 1 were given when no bactericidal activity was detected. (DOCX) [file pone.0139847.s002.docx]

|  | *S.* Typhimurium strain | | | | | | | | | | |  |
| --- | --- | --- | --- | --- | --- | --- | --- | --- | --- | --- | --- | --- |
| *S.* Typhimurium conjugate | SL1344 | LT2 | Ke237 | Ke238 | Ke244 | D24533 | D24545 | Ke249 | D22477 | D25352 | D23580 | Geometric  Mean* |
| 1418 | 3357 | 1917 | 34600 | 6407 | 5437 | 3985 | 4810 | 5636 | 5829 | 1 | 17741 | 2811 |
| 2189 | 19518 | 54167 | 17895 | 5060 | 4396 | 77 | 6849 | 2520 | 10875 | 1 | 8235 | 2673 |
| 2192 | 18571 | 3750 | 9790 | 1 | 1 | 345 | 4847 | 1 | 5656 | 1 | 16395 | 232 |
| D23580 | 2200 | 2778 | 33595 | NA | NA | NA | 3862 | NA | 4705 | NA | 21792 | 6582 |
| SL1344 | 4628 | 8167 | 10115 | NA | NA | NA | 1158 | NA | 2538 | NA | 4671 | 4169 |
| LT2 | 2590 | 625 | 1525 | NA | NA | NA | 1 | NA | 621 | NA | 1569 | 366 |

NA: results not available, experiment not performed.
*values represent the serum titer geometric means relative to each conjugate-serum against all tested strains.
