# Supplementary material for: Strain Selection for Generation of O-Antigen-Based Glycoconjugate Vaccines against Invasive Nontyphoidal Salmonella Disease
Source: PLoS One. 2015 Oct 7;10(10):e0139847. doi: 10.1371/journal.pone.0139847 (PMC4596569; doi:10.1371/journal.pone.0139847)
Supplement: S2 Table — (DOCX) [file pone.0139847.s003.docx]

|  | *S.* Typhimurium strain | | | | | | | | | | | | | |  |
| --- | --- | --- | --- | --- | --- | --- | --- | --- | --- | --- | --- | --- | --- | --- | --- |
| *S.* Typhimurium conjugates | LT2 | SL1344 | D23580 | 1418 | 2189 | 2192 | D22477 | D24533 | D24545 | D25352 | Ke237 | Ke238 | Ke244 | Ke249 | Geometric mean* |
| LT2 | 2845 | 4034 | 1242 | 2169 | 1524 | 421 | 3627 | 3 | 707 | 593 | 353 | 1754 | 1694 | 466 | 806 |
| SL1344 | 2377 | 3543 | 1113 | 1973 | 719 | 127 | 688 | 6 | 479 | 590 | 496 | 3739 | 950 | 208 | 594 |
| D23580 | 700 | 786 | 1282 | 642 | 514 | 207 | 2687 | 219 | 342 | 808 | 179 | 2845 | 725 | 167 | 580 |
| 1418 | 3206 | 4406 | 1963 | 2388 | 2102 | 517 | 2550 | 191 | 1473 | 870 | 700 | 2825 | 3042 | 2866 | 1608 |
| 2189 | 3780 | 5415 | 2883 | 4110 | 2620 | 1036 | 1958 | 2 | 1037 | 930 | 692 | 3238 | 846 | 596 | 1064 |
| 2192 | 2514 | 4681 | 1830 | 3116 | 863 | 152 | 2215 | 2 | 762 | 491 | 353 | 3331 | 1128 | 646 | 736 |
| negative | 2 | 1 | 2 | 2 | 2 | 2 | 5 | 6 | 2 | 2 | 3 | 6 | 2 | 2 | 806 |

*values represent geometric means of fluorescent signals of positive bacteria (excitation: 650 nm, emission: 668 nm)
