# Supplementary material for: Strain Selection for Generation of O-Antigen-Based Glycoconjugate Vaccines against Invasive Nontyphoidal Salmonella Disease
Source: PLoS One. 2015 Oct 7;10(10):e0139847. doi: 10.1371/journal.pone.0139847 (PMC4596569; doi:10.1371/journal.pone.0139847)
Supplement: S3 Table — Bactericidal activity was determined as serum dilutions necessary to obtain 50% percent CFU reduction at T180 compared with T0. Serum titers equal to 1 were given when no bactericidal activity was detected. (DOCX) [file pone.0139847.s004.docx]

|  | *S.* Enteritidis strain | | | | | | | | | |  |
| --- | --- | --- | --- | --- | --- | --- | --- | --- | --- | --- | --- |
| *S.* Enteritidis conjugate | CMCC4314 | D24359 | D24953 | 618 | 502 | IV3453219 | Ke016 | Ke117 | Ke180 | Ke151 | Geometric mean* |
| 502 | 234 | 1 | 1 | 1 | 1 | 1 | 852 | 31 | 445 | 219 | 15 |
| 618 | 950 | 1 | 1 | 1 | 3 | 5 | 5939 | 7103 | 6204 | 6 | 42 |
| IV3453219 | 167 | 1 | 1 | 1 | 1 | 1 | 764 | 487 | 1359 | 12 | 16 |
| D24359 | 153 | 1 | 1 | 1 | 1 | 1 | 2300 | 896 | 367 | 42 | 19 |

*values represent the serum titer geometric means relative to each conjugate-serum against all tested strains.
