# Supplementary material for: Strain Selection for Generation of O-Antigen-Based Glycoconjugate Vaccines against Invasive Nontyphoidal Salmonella Disease
Source: PLoS One. 2015 Oct 7;10(10):e0139847. doi: 10.1371/journal.pone.0139847 (PMC4596569; doi:10.1371/journal.pone.0139847)
Supplement: S4 Table — (DOCX) [file pone.0139847.s005.docx]

|  | *S.* Enteritidis strain | | | | | | | | | | |  |
| --- | --- | --- | --- | --- | --- | --- | --- | --- | --- | --- | --- | --- |
| *S.* Enteritidis conjugates | IV3453219 | Ke151 | Ke180 | Ke016 | Ke117 | D24953 | 502 | 6718 | IV3453219 | D24359 | CMCC4314 | Geometric mean* |
| 502 | 89 | 115 | 177 | 161 | 181 | 190 | 121 | 109 | 156 | 73.2 | 123 | 130 |
| 618 | 359 | 341 | 559 | 324 | 435 | 668 | 688 | 782 | 474 | 312 | 1115 | 507 |
| IV3453219 | 89 | 116 | 159 | 126 | 171 | 185 | 192 | 203 | 141 | 135 | 272 | 156 |
| D24359 | 89 | 144 | 175 | 112 | 231 | 183 | 141 | 193 | 96.1 | 106 | 256 | 148 |
| negative | 2 | 1 | 2 | 2 | 2 | 2 | 5 | 6 | 2 | 2 | 3 | 2 |

*values represent geometric means of fluorescent signals of positive bacteria (excitation: 650 nm, emission: 668 nm)
